# Elaborating the Iodine/Polyiodide Equilibrium Effects in Nanoporous Carbon‐based Battery Electrode via Extreme Mass Asymmetry in Hybrid Cells

**DOI:** 10.1002/celc.202100458

**Published:** 2021-08-18

**Authors:** H. Schranger, S. Khosravi, H. Fitzek, Q. Abbas

**Affiliations:** ^1^ Institute for Chemistry and Technology of Materials Stremayrgasse 09 Graz University of Technology 8010 Graz Austria; ^2^ Graz Centre for Electron Microscopy Steyrergasse 17 8010 Graz Austria

**Keywords:** hybrid energy storage, iodine electrodeposition, nanoporous carbon, supercapacitor, iodide redox-active electrolyte

## Abstract

This article discusses the conversion of electrodeposited iodine to polyiodides within the nanopores of carbon electrodes that affect the performance of iodide electrolyte‐based electrochemical cells. Here, carbon electrodes have been polarized in aqueous sodium iodide electrolyte to store charge in the form of solid iodine via highly reversible reaction (2I^−^⇌I_2_+2e^−^). The stored iodine within the pores interacts with free iodide ions present in the bulk electrolyte via comproportionation reactions leading to polyiodide (I_3_^−^ and I_5_^−^) formations. By tuning the mass asymmetry of carbon electrodes in hybrid cells and using the in‐situ Raman spectroscopy on positive battery electrode, we show the influence of iodine/polyiodides equilibrium shifts on the self‐discharge and voltage rebounds during open circuit conditions. This study provides insights into the charging mechanisms of carbon electrodes for iodine‐based hybrid supercapacitors and battery systems.

## Graphical abstract

**Iodides oxidation within carbon nanopores** forms iodine leading to complete transformation of charge storage from a pure capacitive to battery behaviour. Iodine retention in the pores and preventing its comproportionation reactions is the key to realize stable hybrid supercapacitors and batteries. This article further refines the understanding on how the equilibrium shifts could affect the performance of hybrid cells with iodide‐based electrolytes.

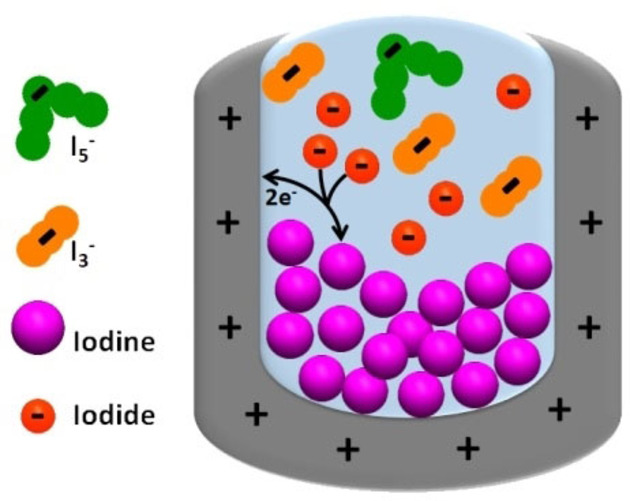

## 1 Introduction

Hybrid electrochemical capacitors are assembled by the coupling of a battery electrode with an electrical double‐layer (EDL) electrode.[^1^, ^2^] Unlike a lithium‐ion hybrid capacitor where lithium intercalated graphite‐based battery electrode operates in an organic electrolyte,[^3^, ^4^] eco‐friendly aqueous iodide‐based hybrid capacitor uses a battery electrode formed due to a highly reversible iodine electrodeposition in the nanopores of carbon.^[5]^ Interestingly, in both the hybrid capacitors, second electrode is made from nanoporous carbon that stores charges at the EDL. Thus, hybridization of a battery‐like electrode with an EDL one results in a high cell capacitance,^[6]^ according to the equation 1.

(1) $$1/C=1/C{}_{-}+1/C{}_{+}$$

Due to its high capacity, the iodine‐charged positive carbon electrode operates in a narrow potential window far below the thermodynamic limit of electrolyte oxidation and consequently experiences reduced ageing effects. It has also been reported that the pore size distribution of the positive carbon electrode plays an important role in accommodating the iodide related redox species.^[7]^ Thus, as a result of iodine electrodeposition within nanoporous carbon, a solid redox electrode is produced. Following this strategy, carbon‐based electrode with electrodeposited iodine was coupled with an EDL electrode to develop a hybrid supercapacitor in a non‐iodide aqueous electrolyte (5 mol L^−1^ NaNO_3_).^[8]^ However, further investigations are required on the electrochemical behavior of iodine containing positive carbon electrode and the effects of comproportionation reactions on its performance.

Recently, it has been shown by using small angle X‐ray scattering (SAXS) that iodine is produced via oxidation of iodide (I^−^) ions in the nano‐confinement of carbon electrode porosity upon polarization as given by equation 2. An iodine pore occupancy of 40 % leading to high packing density has been achieved at much faster rates than intercalation electrodes.^[5]^ These findings clearly show the potential benefits of iodine/carbon chemistry, which could be used to design new batteries with power density comparable to that of hybrid supercapacitors.

(2) $$2I{}^{-}\vec{\leftarrow }I2+2e{}^{-}$$

**Scheme 1:**
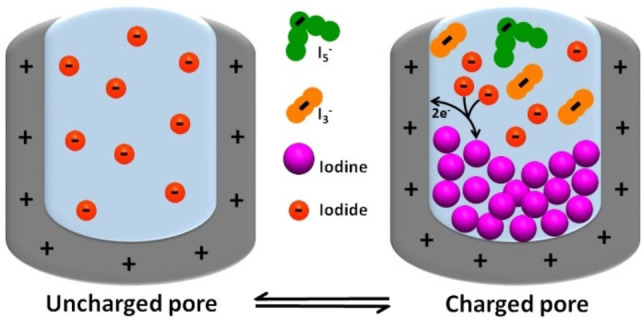
Electrodeposition of iodine from oxidation of iodides in a charged pore containing iodide‐based electrolyte, iodine is converted to triiodides (I_3_^−^) via comproportionation reaction with excess of iodides in the bulk electrolyte, followed by the formation of pentaiodides (I_5_^−^).

In situ Raman spectroscopy confirmed that once iodine is produced inside the pores, it then converts to polyiodides (I_3_^−^ and I_5_^−^) via comproportionation reactions under the influence of excess I^−^ present in the bulk aqueous electrolyte.^[5]^ The polyiodides progressively move out of the electrode porosity into the bulk electrolyte and also towards the negative EDL electrode triggering the so‐called shuttle‐effect. The shuttling of I_3_^−^ and I_5_^−^ is the main reason of performance loss in these hybrid supercapacitors. One way to properly store iodine and to tackle the issue of shuttling is to use carbons with different pores.[^9^, ^10^, ^11^] Due to very fast iodide/iodine redox reaction, there is also a high risk of polyiodides generation – as soon as iodine is electrodeposited under electrochemical polarization, it is converted to I_3_^−^ and I_5_^−^.^[12]^ According to the equations 3 and 4 and as shown in scheme 1, an equilibrium tends to establish between the solid iodine and the polyiodides in presence of free iodide ions in the electrolyte.[^13^, ^14^, ^15^]

(3) $$I{}_{2}+I{}^{-}\vec{\leftarrow }I{}_{3}{}^{-}$$

(4) $$I{}_{3}{}^{-}+I{}_{2}\vec{\leftarrow }I{}_{5}{}^{-}$$

Here, we look at two hybrid cells with completely different active carbon mass ratio in iodide‐based aqueous electrolyte. To show, how the iodine/polyiodide equilibrium influences the electrochemical characteristics of a hybrid cell, extreme mass asymmetry of electrodes has been employed. The effects of working electrode (WE) to counter electrode (CE) mass ratio have been discussed by comparing hybrid cells with WE : CE=1 : 100 and WE : CE=1 : 2 (increased amount of active carbon in CE than WE) while using 1 mol L^−1^ NaI aqueous electrolyte. The formation of I_3_^−^ and I_5_^−^ as a result of comproportionation reaction is controlled by the iodine, the interactions of which also bring structural changes in the carbon lattice. Furthermore, the influence of active mass asymmetry of electrodes on the hybrid cell performance (self‐discharge and potential rebound behavior during an open circuit period) that imitates the iodine/carbon electrode in a battery cell has been discussed.

## 2 Results and Discussion

The electrochemical performance of two hybrid cells has been compared in aqueous sodium iodide (1 mol L^−1^ NaI) electrolyte. This discussion is supported by the in situ Raman spectroscopy investigations where polyiodides formation, their movement out of the pores as well as resulting modifications in the carbon structural parameters has been discussed. The hybrid cells were polarized up to a low voltage limit of 0.4 V or 0.6 V, in order to essentially study the charge/discharge mechanisms of electrodes without involving faradaic reactions related with electrolyte oxidation and reduction.

### 2.1 Performance of Individual Electrodes in Hybrid Cells

**Figure 1:**
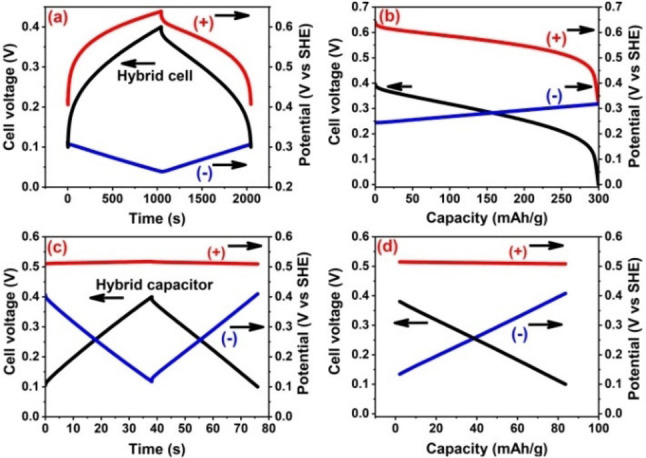
Galvanostatic charge/discharge curves of positive and negative carbons electrodes for hybrid cells with WE : CE=1 : 100 (a, b) and WE : CE=1 : 2 (c, d) in a two‐electrode cell equipped with a reference electrode using 1 mol L^−1^ NaI charged between a cell voltage of 0.1 to 0.4 V at 0.2 A g^−1^.

The galvanostatic charge/discharge data of hybrid cells and the individual electrode potential profiles are presented in Figure 1. Both cells with mass ratio WE : CE=1 : 100 and WE : CE=1 : 2 were polarized in 1 mol L^−1^ NaI between 0.1 and 0.4 V. In figures 1a, b for hybrid cell with WE : CE=1 : 100, the positive electrode's charging behavior translates into the overall cell behavior. The potential ranges of positive and negative electrodes are 205 mV and 59 mV respectively. This is explained by the large size of counter electrode (high active mass) which stores enormous amount of charges at the EDL and operates in relatively small potential window with linear charge/discharge. To balance the charges on the big counter electrode, the positive electrode has limited available porosity. Hence, despite the high amounts of iodine electrodeposition, the capacity is limited by the size of working electrode (less surface and pores availability). A larger counter electrode necessitates the charges to be balanced at the positive battery‐like electrode. The EDX data in Figure S1 confirms the presence of iodine in carbon electrode after polarization in sodium iodide electrolyte. The presence of mesopores in the working electrode (see Figure S2 in supplementary information) favors the iodine electrodeposition.

Next, comparison is drawn between the above described highly asymmetric hybrid cell with the one having mass ratio WE : CE=1 : 2. The later one is a hybrid capacitor by charge storage mechanisms, but almost symmetric by electrodes mass and certainly symmetric by the type of carbon material used. This hybrid cell is polarized up to a similar voltage of 0.4 V and one can see a clear difference in the shape of charge/discharge curves (Figure 1c, d). Hybrid cell with 1 : 2 mass ratio displays overall symmetric charge/discharge curves which is characteristic of the negative EDL electrode. In this case, the EDL negative electrode stores charges at the EDL and operates in a large potential window of 274 mV with symmetric charge/discharge curves. Such large potential window of the negative electrode is driven by the high‐capacity positive electrode where charges are stored via iodine electrodeposition. The positive electrode operates in a very narrow potential window (8 mV) and compared to the size of the counter electrode, the positive electrode possess high iodine capacity and probably a part of its porosity is still vacant.

The negative electrode in the WE : CE=1 : 2 case operates down to 0.1 V vs. standard hydrogen electrode (SHE), which is lower than 0.21 V vs. SHE for the highly asymmetric hybrid cell. Nevertheless, the potential limits of positive and negative electrodes in both the hybrid cells are within the thermodynamics stability window of water (∼1.23 V). It is noteworthy that the shape of charge/discharge curves of hybrid cell with 1 : 2 mass ratio is determined by the negative electrode which also determines the overall capacitance. The charge/discharge curve of hybrid cell of electrodes mass ratio 1 : 2 is truly like a supercapacitor, and it has been described previously that with appropriate mass balancing a hybrid supercapacitor could be realized.^[15]^ The difference of capacities between the two hybrid cells is obvious, the cell with 1 : 100 mass ratio exhibits discharging capacity of 300 mAh g^−1^ versus charging capacity of 340 mAh g^−1^ while the cell with 1 : 2 mass ratio displays discharge capacity of 80 mAh g^−1^ versus charging capacity of 84 mAh g^−1^. Overall, these findings are in line with the equation 1, where charging behavior of the hybrid cell is determined by the electrode with low capacitance.

### 2.2 Monitoring the Evolution of I_3_^−^ and I_5_^−^ via in situ Raman Spectroscopy

**Figure 2:**
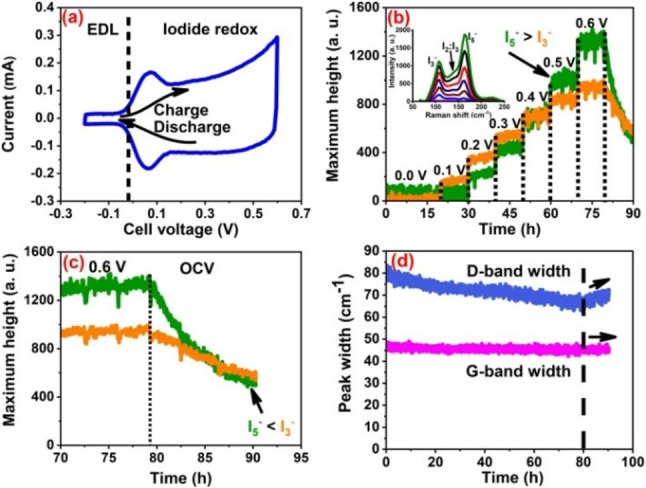
In situ Raman spectroscopy investigation of a hybrid cell using 1 mol L^−1^ NaI with WE : CE=1 : 10 to monitor polyiodides and carbon electrode parameters: cyclic voltammogram at 0.08 mV s^−1^ (a), I_3_^−^ and I_5_^−^ evolution intensities during hold at different values of potential (b, c), and G‐ and D‐band width (d), during total period of potential hold of 80 hours and 10 hours of open circuit potential after polarizing to 0.6 V.

Cyclic voltammogram in Figure 2a is recorded with an in situ Raman cell using 1 mol L^−1^ NaI between a voltage window of −0.2–0.6 V at 0.08 mV s^−1^. A low discharge cut‐off voltage down to −0.2 V was selected to charge both electrodes as typical EDL. During a positive voltage sweep, however, the positive electrode's charge storage mechanism changes to a battery‐like as discussed in detail for Figure 1. The presence of a large counter electrode which stores charges at the EDL drives the positive carbon electrode to charge via iodine electrodeposition in the region of cell voltage from 0.05 V to 0.6 V. Obviously, the iodide/iodine redox potential of +0.54 V vs. SHE is close to the cell equilibrium potential (also called potential at zero voltage). A redox potential closer to the cell equilibrium potential entails the use of iodine redox activity for the entire selected voltage range. As discussed for Figure 1c, the overall shape of charge/discharge curve is determined by the negative electrode, and a square‐like CV in Figure 2a verifies the previous finding.

Polyiodides evolution was studied during in situ Raman investigations (Figure 2b, c) under potential hold test from −0.2 to 0.6 V. Starting from −0.2 V and 0.0 V, which is the EDL region, no signals for polyiodides were observed (inset in Figure 2b). Upon further potential hold at 0.1 V, I_2_ starts to produce leading to the formation of I_3_^−^. The conversion rate of iodine to I_3_^−^ is fast in the beginning until reaching 0.4 V. The shift in the intensity of I_3_^−^ and I_5_^−^ indicates that until 0.4 V, most of the iodide is oxidized to iodine. As the concentration of free iodide in bulk electrolyte drops, so does the intensity of I_3_^−^ which continues to be converted to I_5_^−^. Afterwards, the I_5_^−^ increases and maintains higher intensity than I_3_^−^ during potential hold at 0.6 V. Similar trend of I_3_^−^ and I_5_^−^ evolution during cyclic voltammetry at low sweep rate has been reported previously.^[5]^ The total potential hold period of 80 hours is followed by an open circuit voltage for 10 hours. During this OCV period, a sudden decay of polyiodides is observed where I_5_^−^ degenerates faster than I_3_^−^. Here, two processes are at play, i) both I_3_^−^ and I_5_^−^ diffuse away into the bulk electrolyte and ii) I_5_^−^ disproportionate to I_3_^−^ and I_2_. The equilibrium which shifted towards I_5_^−^ than I_3_^−^ during potential holds, shifts back to iodine during OCV. The effect of these disproportionation conversions on the hybrid cell performance will be further discussed in next sections.

The trends for G‐band and D‐band in Figure 2d and Figure S4 indicating changes in carbon structural parameters are similar as reported in our previous works.[^5^, ^16^] The downshift of D‐band is attributed to charge transfer between carbon and iodide/iodine. Since iodides are electron rich and iodine is deficient in electron, a charge transfer in both directions (to‐ and from carbon) causes net effect in terms of D‐ and G‐band downshift and narrowing. A relatively small change in G‐band may be due to the net effect of two factors working in opposite direction i) iodine buildup inside the pores causing strain and ii) the charge transfer from iodides to carbon electrode. Nevertheless, all the carbon related parameters are gradually restored during open circuit which indicates the reversible nature of charge transfer and consequent structural changes in carbon lattice. This data also highlights the subtlety of carbon/iodide interaction and its reversibility during long term cycling or potential hold ageing tests. To further assess the electrochemical behavior of electrodes during an open circuit condition, self‐discharge and potential rebound tests are described in next sections.

### 2.3 Self‐discharge Investigations of Hybrid Cells and Individual Electrodes

**Figure 3:**
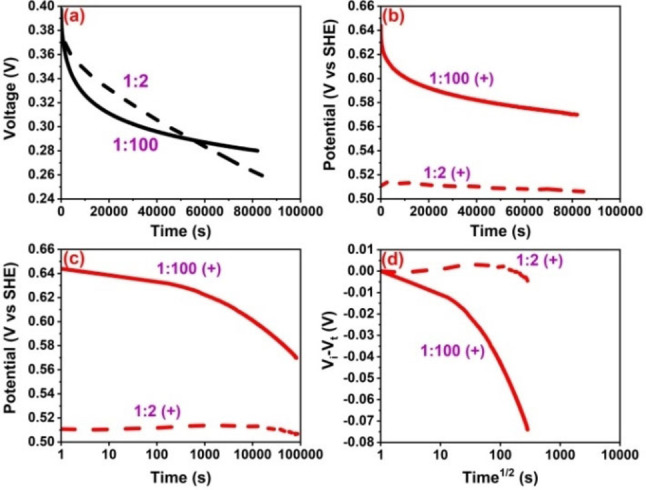
Self‐discharge of hybrid cells (a), electrodes in a two‐electrode cell setup with a reference electrode and WE : CE=1 : 100 (full line) and 1 : 2 (dashed line) in aqueous solution of 1 mol L^−1^ NaI after a potential hold of 4 h at 0.4 V (b), voltage versus log t (c) and V_i_‐V_t_ versus t^1/2^ (d).

The high asymmetry of the carbon electrodes active mass in WE : CE=1 : 100 enables us to understand the individual electrode charging/discharging behavior in comparison to the hybrid cell with WE : CE=1 : 2 mass ratio of electrodes. The self‐discharge behavior of two electrode cells is compared in Figure 3a. The pattern of fast voltage decay after a hold in hybrid cell 1 : 100 is similar to the positive electrode behavior in Figure 3b, while for the hybrid cell 1 : 2, the voltage decay seems to be replica of the negative electrode behavior in Figure S6a. Therefore, one can infer that just like the hybrid cell capacitance is determined by the electrode with lower capacitance (equation 1), the open circuit voltage profiles follow a similar pattern and they mainly replicate the potential profile of electrode with lower capacitance. A relatively quick voltage decay for hybrid cell 1 : 100 for 50000 seconds before reaching to some sort of plateau can be correlated to situation discussed in Figure 2b,c. Here, the electrodeposited iodine is converted to polyiodides which then diffuse into the bulk electrolyte and some part of it may disproportionate leading to sharp voltage decay just after the hold. As soon as the iodide species reach to equilibrium, the voltage drops less sharply and reaches to a nearly steady state. Self‐discharge of positive battery‐like electrode in iodide/iodine redox system has been previously reported,^[17]^ which, from the point of view of electrode mass balancing, was a similar case to the one reported here (WE : CE=1 : 2). For less mass asymmetric hybrid cell, the positive electrode displays a very low self‐discharge owing to the immobilization of iodine‐related species. This behavior has been dedicated to strong interactions of iodide/iodine species with the positive carbon electrode, which do not easily diffuse out. The self‐discharge at the negative electrode for 1 : 2 hybrid cell might be controlled by the charge redistribution effect, which is a typical behavior for an EDL electrode.^[18]^

Further, positive electrodes in both hybrid cells display a slop which is relatively higher for the hybrid cell with electrode mass ratio of WE : CE=1 : 100 (Figure 3c). This indicates some kind of interactions between the iodide species and the carbon electrode surface. Two slopes in V_i_‐V_t_ vs time curves (Figure 3d) suggest two different processes, one which is under the influence of diffusion of iodide species into the bulk electrolyte and towards the negative electrode (diffusion‐controlled mechanism) and the second slop due to the large negative electrode leading to fast disproportionation of polyiodide species. At the same time, the interactions of iodide/iodine species with the negative electrode surface lead to high self‐discharge (see Figure S6). By contrast, a less prominent slop for the potential profile of positive electrode in hybrid cell with mass ratio of WE : CE=1 : 2, indicates less effect of diffusion‐controlled processes. Clearly, in this case, the positive electrode works in a constant potential range, and the disproportionation conversion of polyiodides may be limited by the relatively low amount of charges stored on the counter (negative) electrode. Therefore, a proper balancing of charges at the working electrode minimizes fast self‐discharge. Overall, self‐discharge is controlled by the diffusion of species in hybrid cell WE : CE=1 : 100 and by activation processes in hybrid cell WE : CE=1 : 2. To further correlate the electrochemical behavior of carbon/carbon hybrid cell with the polyiodides diffusion and inter‐conversion, potential rebound tests have been presented in next section.

### 2.4 Potential Rebound of Electrodes at Different Cut‐off Cell Voltages

**Figure 4:**
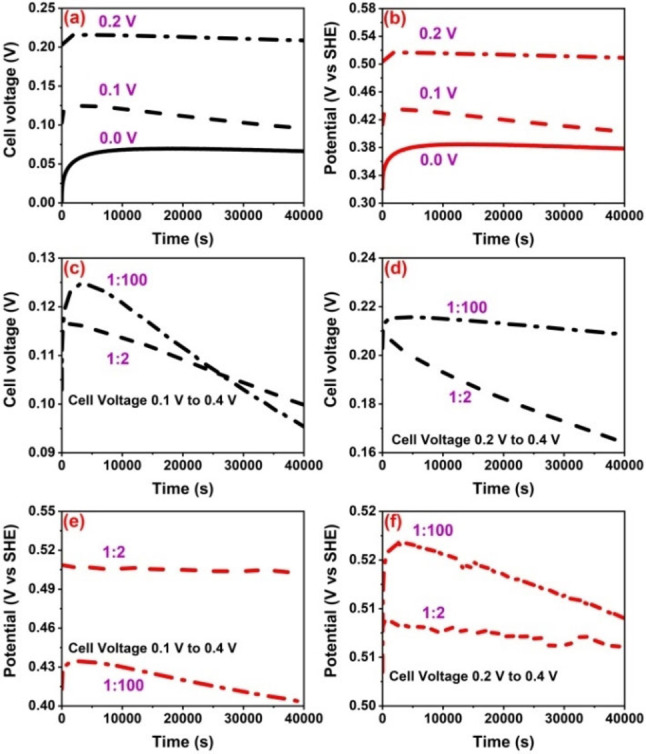
Voltage rebound of hybrid cell (a) and positive electrode (b) with 1 mol L^−1^ NaI and mass ratio WE : CE=1 : 100 after being discharged to 0.0 V, 0.1 V and 0.2 V, comparison of voltage rebound profiles between two hybrid cells at cut‐off voltage 0.1 V (c) and 0.2 V (d), and potential rebound of positive electrodes in cells with mass ratio of WE : CE=1 : 100 (dashed line) and WE : CE=1 : 2 (dashed dotted line) discharged down to a minimum voltage of 0.1 V (e) and 0.2 V (f).

Voltage rebound of hybrid cell with electrodes mass ratio WE : CE=1 : 100 where it was discharged to 0.0 V, 0.1 V and 0.2 V after charging up to 0.4 V, is shown in Figure 4a. When discharging down to 0.0 V, a maximum rebound of cell voltage is observed and this can be attributed to the high amount of charges distributed in the positive electrode matrix. In other words, the self‐charging of positive electrode is driven by the charge distributed into the deep porosity of the carbon electrode driving a backward I_3_^−^ and I_5_^−^ diffusion. This is proven by the fact that as the cut‐off voltage is increased gradually, a decrease of voltage rebound effect at the onset is observed. Considering the hybrid cell voltage rebound is replica of the positive electrode behavior (Figure 4b), it verifies the previous discussion that electrode with low capacitance determines the overall electrochemical behavior of the hybrid cell.

Hence, the voltage rebound in case of WE : CE=1 : 100 is determined by the potential rebound of positive battery electrode, while it is controlled by the negative electrode in case of WE : CE=1 : 2 hybrid cell (Figure S7a). Before setting up a voltage rebound, a specific negative current was applied on the hybrid cell to discharge it fully or partially. At the point of discharge, polyiodides are consumed by iodine formation which converts to iodide ions. When the voltage reaches to 0.0 V, more iodides move towards the surface while charge redistribution effect is still in play – charge is still present in the deep porosity of electrode which needs to be balanced. Consequently, after a discharge to 0.0 V, the cell voltage rebounds in absence of an applied current, and at this point, iodides conversion to iodine may be controlled by the charge distributed in the electrode. In this context when positive electrode is discharged to low potentials, a stronger potential rebounds occurs for WE : CE=1 : 100, which is driven by the large counter electrode. On the other hand, The extent of backward diffusion of polyiodides and consequently the self‐charging of positive electrode is less in case of hybrid cell with nearly symmetric active mass of electrode in WE : CE=1 : 2. Overall, the data on the individual electrodes of hybrid cells shows the tendency of the hybrid cell to equilibrate after being fully discharged to a selected minimum cell voltage, where positive electrode controls the voltage profile for WE : CE=1 : 100 and negative electrode determines the behavior of WE : CE=1 : 2.

## 3 Conclusions

The nanoporous carbon‐based electrode charged with solid iodine losses its high capacity under the influence of bulk electrolyte free‐iodides that form polyiodides (I_3_^−^ and I_5_^−^). Exceedingly high amount of I_5_^−^ and I_3−_ are formed that tend to reach to equilibrium with solid iodine at open circuit conditions. This phenomenon is more pronounced in highly mass asymmetric hybrid cell where the battery electrode displays self‐discharge controlled by the diffusion of polyiodide species. Despite the fact that more solid iodine is formed in such a cell, equilibrium shifts cause comproportionation reactions to be fast. On the negative EDL electrode, activation‐controlled mechanisms are mainly due to the interactions of iodides with the carbon surface. Release of polyiodides in iodide‐rich electrolyte necessitates the shifting of iodinated battery electrode to an iodide‐free electrolyte (e. g., aq. NaNO_3_) with an objective to shift equilibrium towards iodine (equation 3) during open circuit period. Iodine/iodide redox potential is close to the hybrid cell equilibrium potential which gives this system an advantage over e. g., bromine or hydroquinone‐based electrolytes. However, a careful manipulation of iodine charged carbon electrode is required when coupling with another capacitive or battery electrode. Then an iodide‐free electrolyte is a preferred option which could help to retain the iodine/iodide reaction within electrode pores and prevent their undesired shuttling. In a wider perspective, these findings will help to predict the electrochemical behavior of iodine‐charged electrode to be coupled with zinc, magnesium, or a carbon‐based anode.

## Experimental Methods

All electrodes used in this study were prepared in free‐standing form by mixing 90 wt % activated carbon (MSP‐20, Kansai Coke and Chemicals), 5 wt % carbon black (C65, Imerys) and 5 wt % polytetrafluoroethylene (60 % dispersion in water from 3 M Chemicals) in isopropanol. The mixture was stirred at 70 °C until the solvent was evaporated. The resulting dough was pressed and rolled with a custom‐built calendering machine to a thickness of 150 μm and dried at 120 °C to evaporate water and the solvent adsorbed in the pores. Sodium iodide (Alfa‐Aesar) based aqueous electrolyte was used at a concentration of 1 mol L^−1^ with pH=6.5 and conductivity=82 mS cm^−1^.

Hybrid cells were constructed in Swagelok‐type three‐electrode setup with an Ag/AgCl reference electrode. The diameter of carbon electrodes was 10 mm which were separated by a glassy fiber GFA separator of thickness=260 μm. To ensure an excess of aqueous electrolyte, three separators in stacked form were used in hybrid cells. Before each self‐discharge measurement, cell was held at a constant voltage for 4 h, while voltage rebound tests were performed directly after discharge to a minimum value. Electrochemical measurements on hybrid cells were performed with a VMP3 multichannel potentiostat/galvanostat (Bio‐Logic Instruments).

In situ Raman spectroscopy was carried out using a LabRAM HR 800 spectrometer combined with an Olympus BX41 microscope, using an in situ cell ECC‐Opto‐Gas from EL‐CELL (Hamburg, Germany), and tested with a single channel SP‐150 (Bio‐Logic Instruments). The spectra were measured from the top of the cell, with the focus plane underneath the standard microscope glass slip (covering the cell assembly) and the electrolyte film on top of the carbon electrode. As an objective lens a 40× Olympus LUCPlanFL N (NA=0.6; corrected for the cover thickness) was used. The laser with 532 nm wavelength was used at a low power of 0.5 mW to avoid any sample alterations. In addition, the DuoScan System was used to continuously scan the laser spot over a 20×20 μm area during potential hold tests and OCV. A spectral (pixel) resolution of about 3.6 cm^−1^ was achieved with the used grating/slit setup (300 mm^−1^; 200 μm). The total acquisition time per spectrum was 120 s (4 accumulations×30 s). The deconvolution of the G‐ and D‐bands was done according to the method by Ferrari et al.^[18]^

## Conflict of interest

The authors declare no conflict of interest.

## Supporting information

As a service to our authors and readers, this journal provides supporting information supplied by the authors. Such materials are peer reviewed and may be re‐organized for online delivery, but are not copy‐edited or typeset. Technical support issues arising from supporting information (other than missing files) should be addressed to the authors.

Supporting Information
