# Supplementary material for: Elaborating the Iodine/Polyiodide Equilibrium Effects in Nanoporous Carbon‐based Battery Electrode via Extreme Mass Asymmetry in Hybrid Cells
Source: ChemElectroChem. 2021 Aug 18;8(16):3155–60. doi: 10.1002/celc.202100458 (PMC8457216; doi:10.1002/celc.202100458)
Supplement: Supplementary file 1 — Supporting Information [file CELC-8-3155-s001.pdf]

ChemElectroChem

Supporting Information

Elaborating the Iodine/Polyiodide Equilibrium Effects in Nanoporous Carbon-based Battery Electrode via Extreme Mass Asymmetry in Hybrid Cells

H. Schranger, S. Khosravi, H. Fitzek, and Q. Abbas*

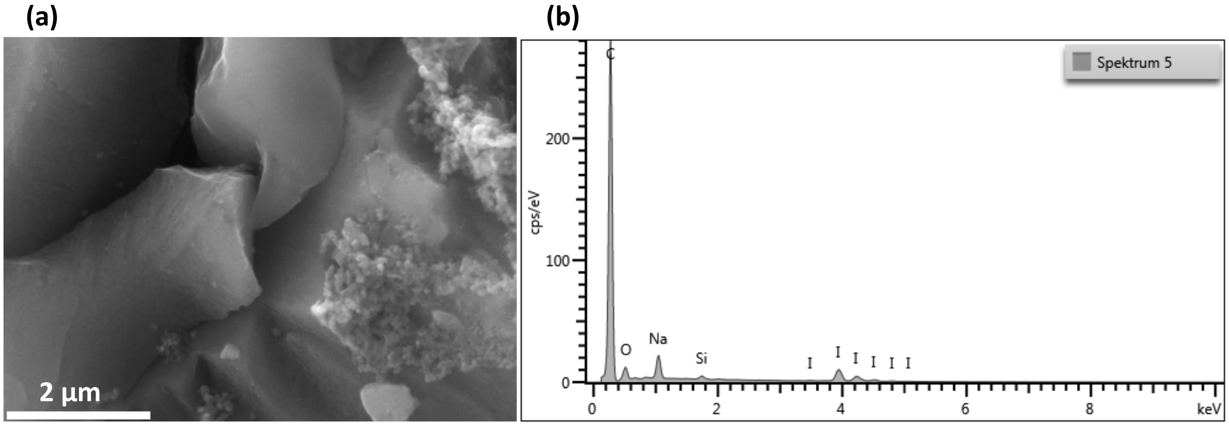

Figure S1. Scanning electron microscopy (a) and EDX data (b) of MSP-20 carbon-based electrode after being electrochemically polarized in 1 mol L^{-1} NaI.

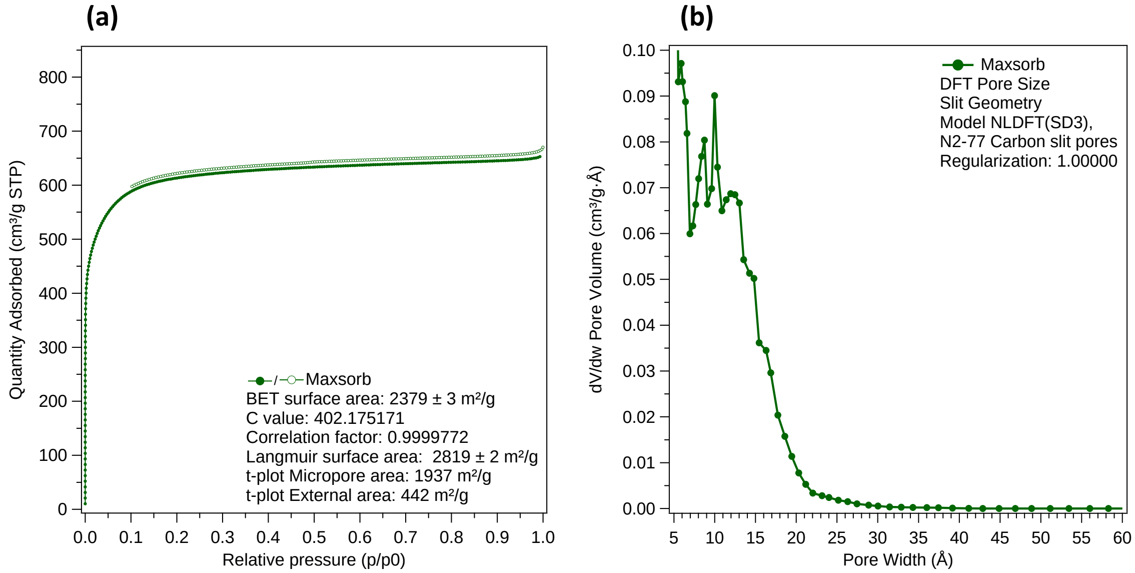

Figure S2. Gas adsorption data of MSP-20 carbon, N_2 adsorption isotherm at 77 K (a) and pore size distribution based on NLDFT model (b).

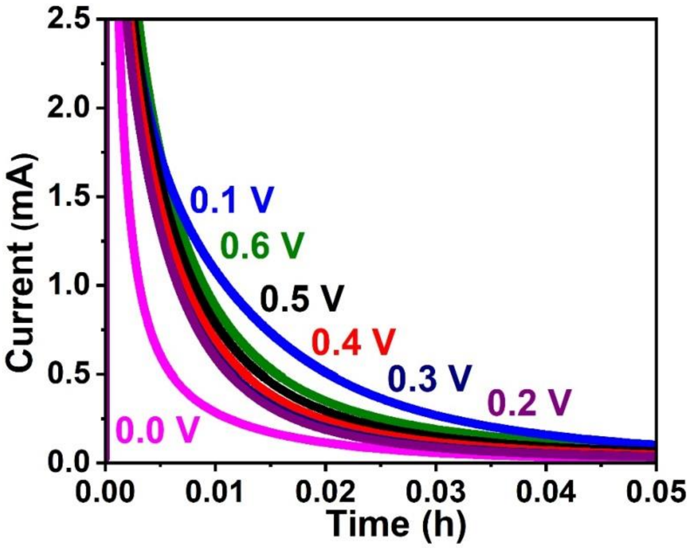

Figure S3. Leakage current profiles during potential holds at various values for an in situ Raman cell using MSP-20 carbon-based electrodes and 1 mol L^{-1} NaI.

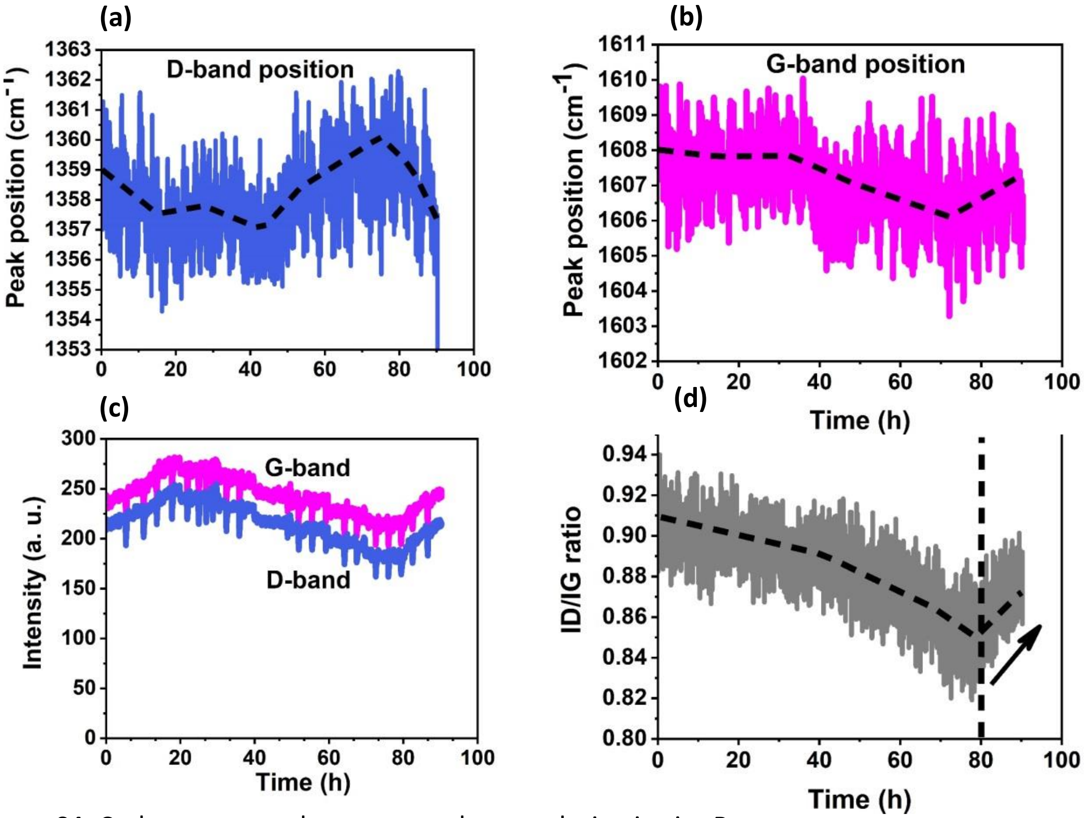

Figure S4. Carbon structural parameter changes during in situ Raman spectroscopy measurements, D-band position (a), G-band position (b), D- and G-band intensity (c), I_D/I_G ratio (d).

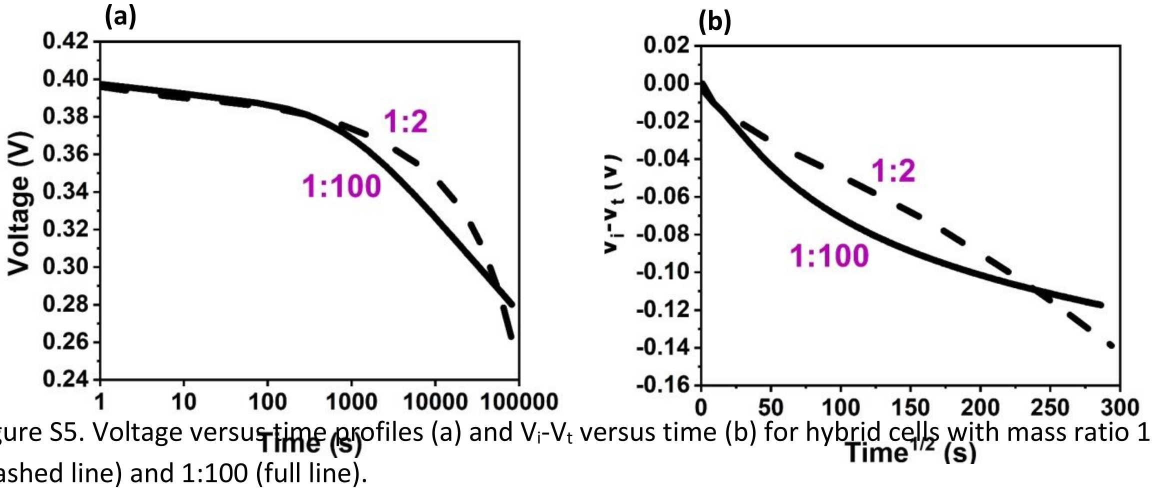

Figure S5. Voltage versus time profiles (a) and V_i-V_t versus time (b) for hybrid cells with mass ratio 1:2 (dashed line) and 1:100 (full line).

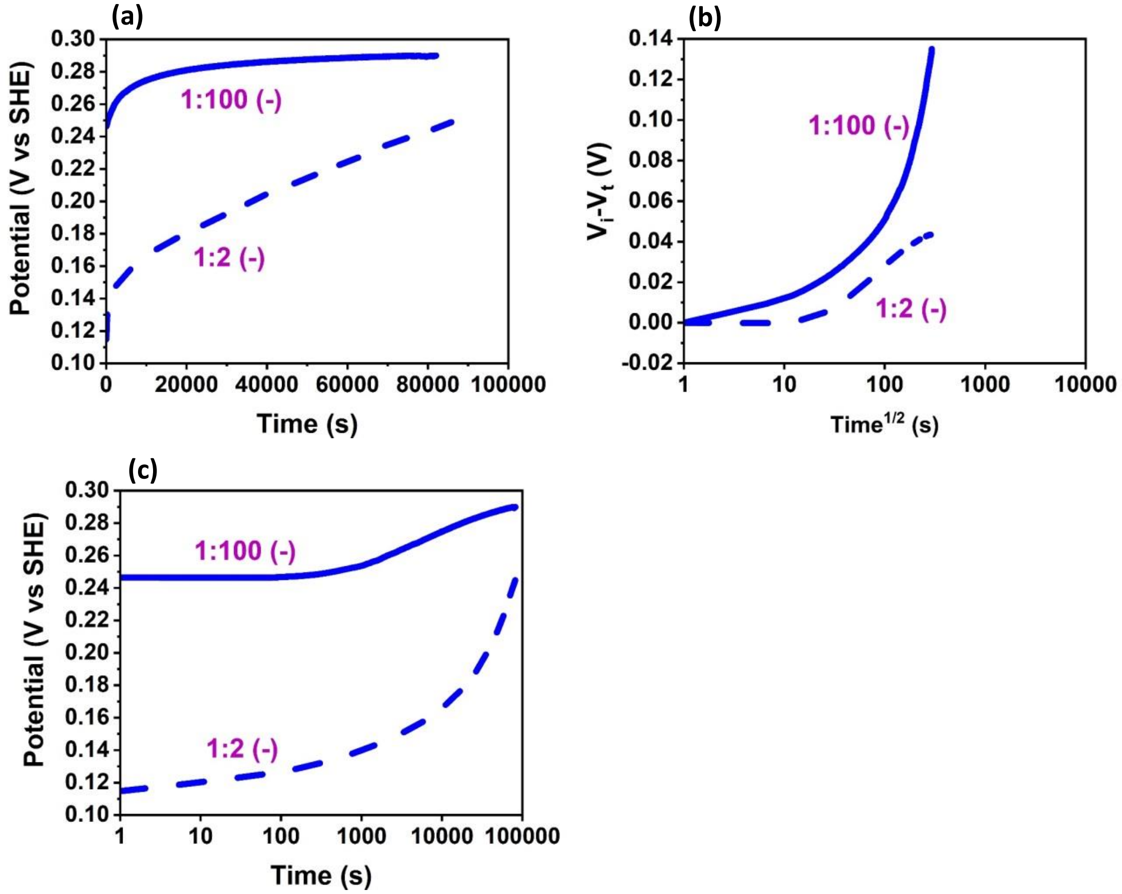

Figure S6. Self-discharge behavior of negative electrodes in hybrid cells 1:2 (dashed line) and 1:100 (full line) mass ratio of positive to negative electrode.

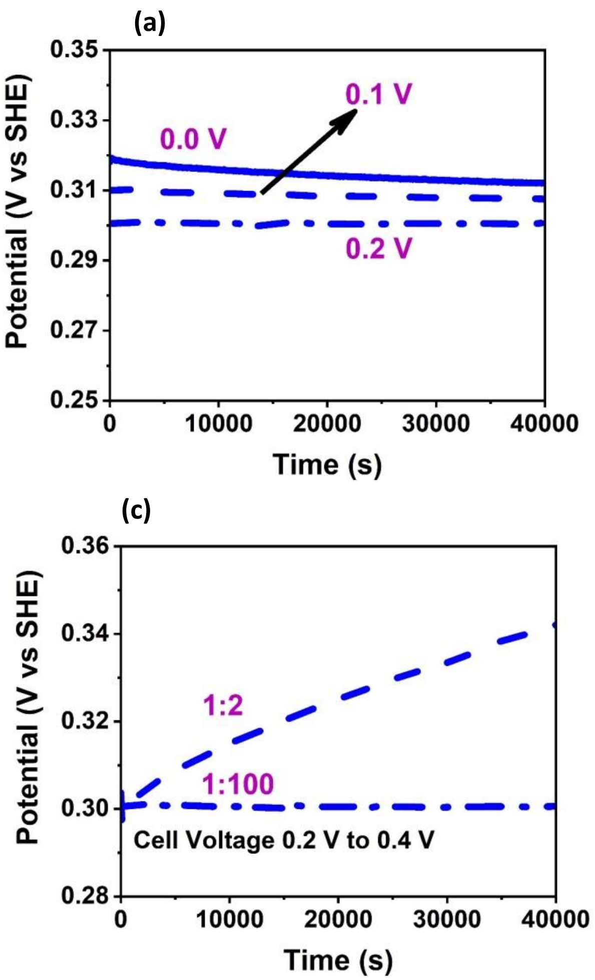

Figure S7. Voltage rebound of negative electrodes for hybrid cell 1:100 (a), and comparison of potential rebound of negative electrodes between hybrid cells 1:2 (dashed line) and 1:100 (dashed-dotted line) discharged down to 0.1 V (b) and 0.2 V (c).
